# Supplementary material for: Comprehensive molecular comparison of BRCA1 hypermethylated and BRCA1 mutated triple negative breast cancers
Source: Nat Commun. 2020 Jul 27;11:3747. doi: 10.1038/s41467-020-17537-2 (PMC7385112; doi:10.1038/s41467-020-17537-2)
Supplement: Supplementary file 1 — Supplementary Information [file 41467_2020_17537_MOESM1_ESM.pdf]

Supplementary Information

Comprehensive molecular comparison of *BRCA1* hypermethylated and *BRCA1* mutated triple negative breast cancers

Glodzik et al.

This document contains:

Supplementary Figure 1. CONSORT diagram.

Supplementary Figure 2. *BRCA1* mRNA expression.

Supplementary Figure 3. Gene expression subtypes.

Supplementary Figure 4. Expanded copy number analyses.

Supplementary Figure 5. Unsupervised gene expression analysis of immune cell type related genes.

Supplementary Methods. Document describing in detail patient selection, experimental procedures, and statistical analyses.

Supplementary References. References for the supplementary methods.

.

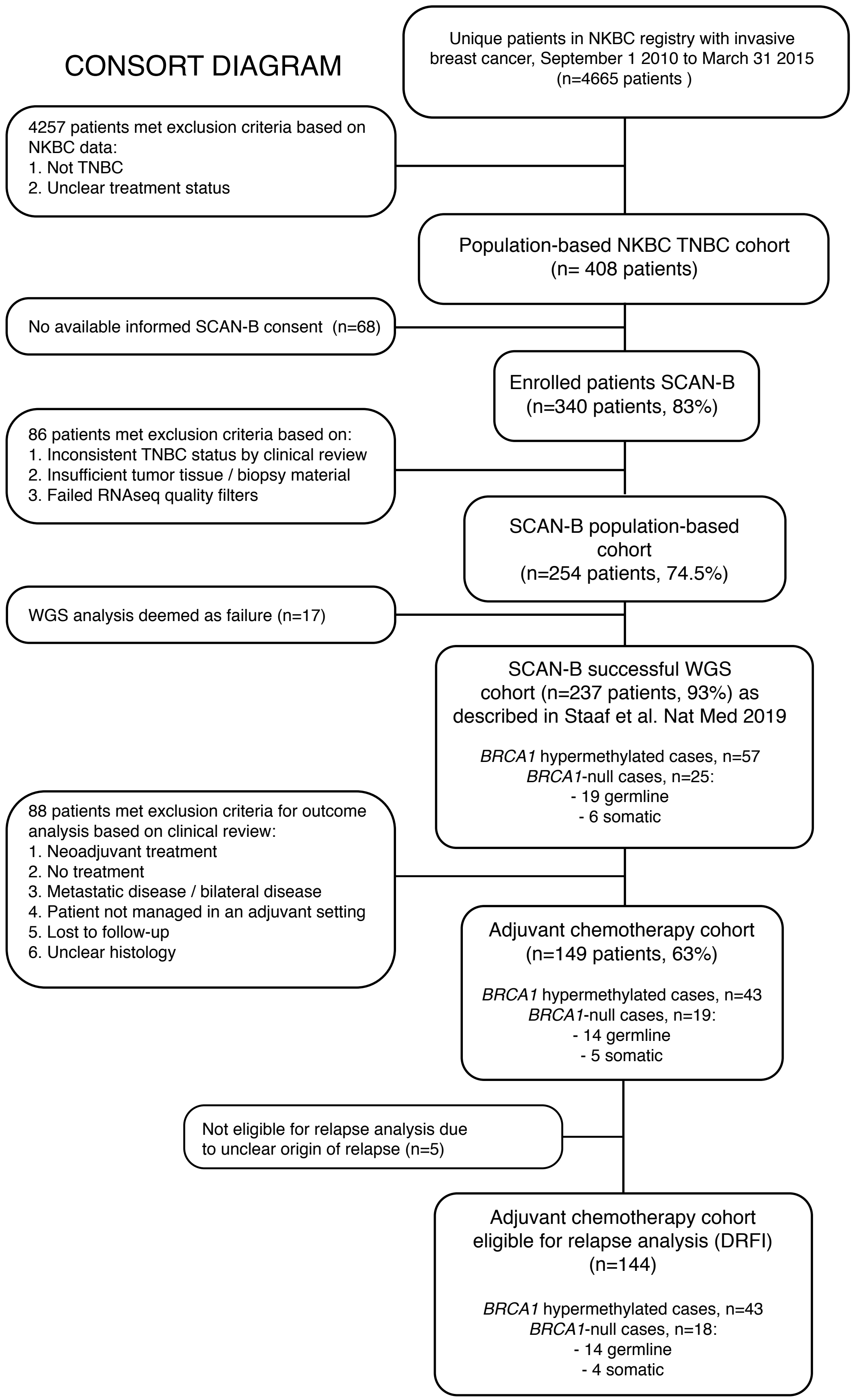

Supplementary Figure 1. CONSORT diagram of inclusion and exclusion steps for deriving the cohort of 237 SCAN-B TNBC cases from the Skåne healthcare region from which *BRCA1* hypermethylated cases were identified based on pyrosequencing. Explicit patient inclusion and exclusion criteria are reported in Staaf et al. Nat Med 2019.

A)

B)

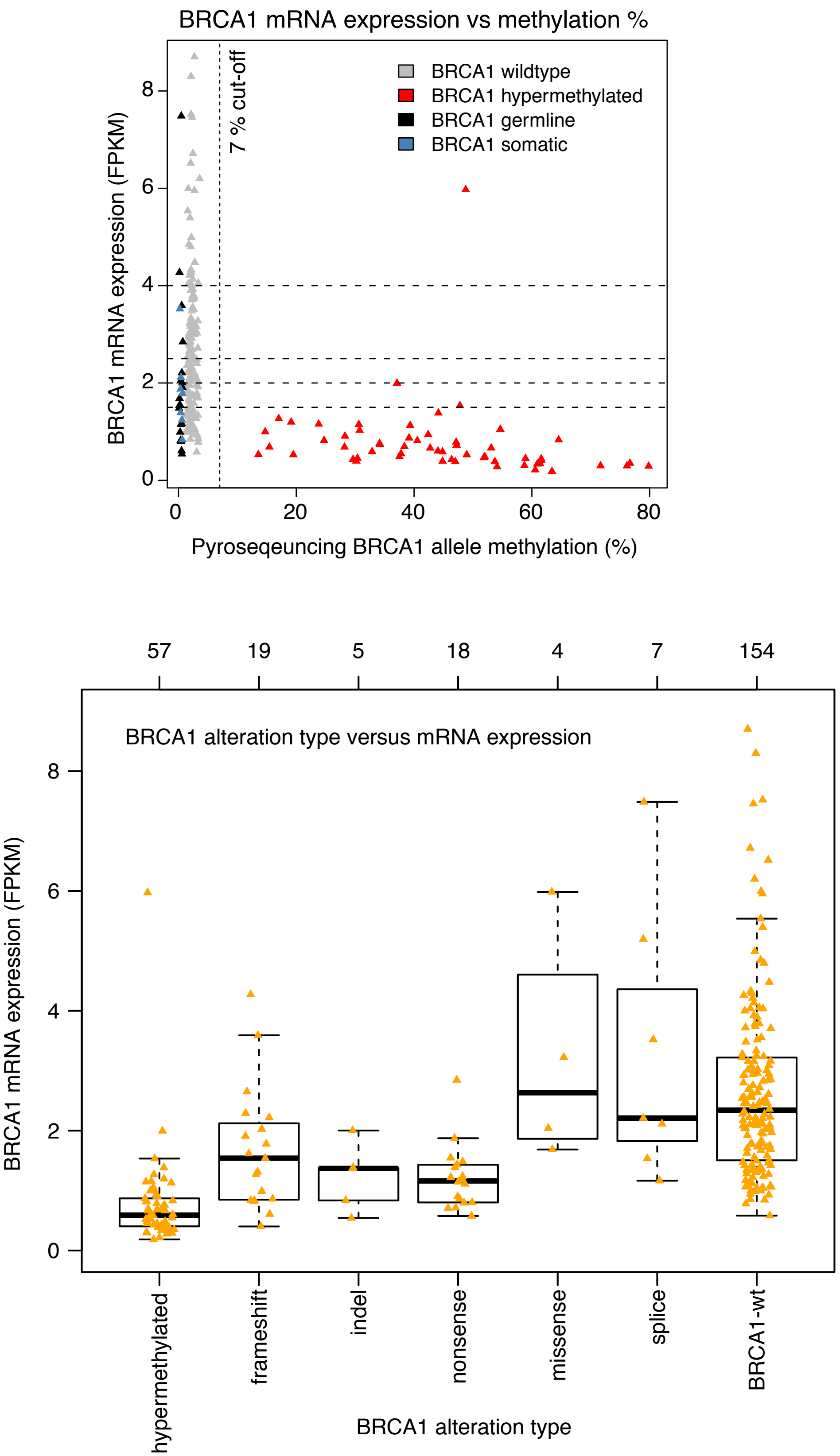

Supplementary Figure 2. (A) *BRCA1* mRNA levels (FPKM) versus pyrosequencing methylation, with sample coloring according to BRCA1 status for 237 SCAN-B cases. **(B)** *BRCA1* mRNA expression (FPKM) for hypermethylated cases, cases with *BRCA1* variants grouped according to mutation type, and cases without *BRCA1* alterations (BRCA1 wt) for the merged set of SCAN-B cases (n=237) and additional *BRCA1*-mutant cases from Jönsson et al. (Cancer Res 2012, n=27). Top axis indicates number of cases per group.

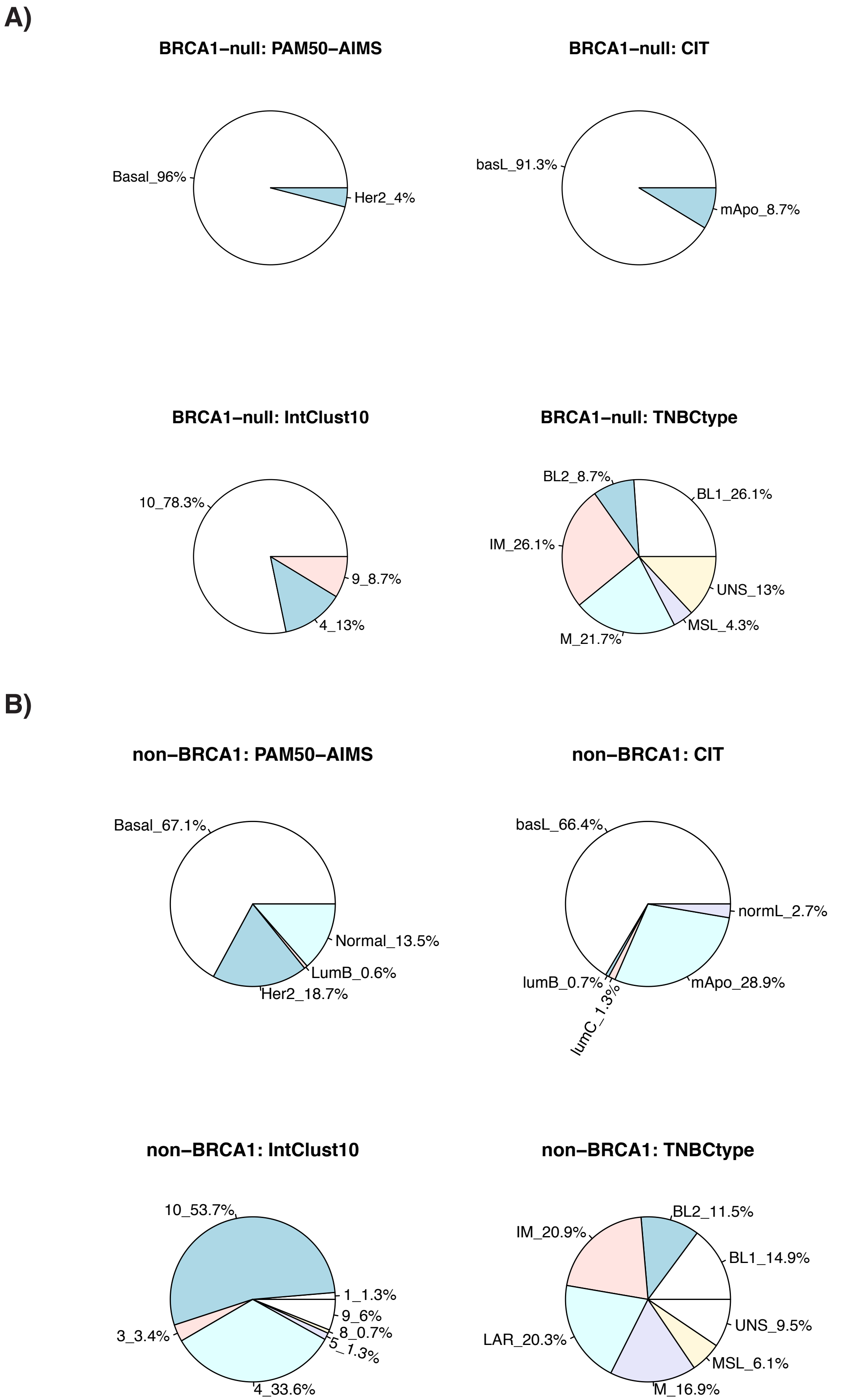

Supplementary Figure 3. Gene expression molecular subtype proportions in BRCA1-null and non-BRCA1 altered SCAN-B cases. The latter group correspond to patients whose tumors are negative for hypermethylation or germline/somatic BRCA1 inactivating variants. Proportions are for all cases belonging to a group from the total 237-sample SCAN-B cohort. (**A**) BRCA1-null cases (n=25). (**B**) non-BRCA1 cases (n=155). Subtype names are explained in Table 2 in the main study.

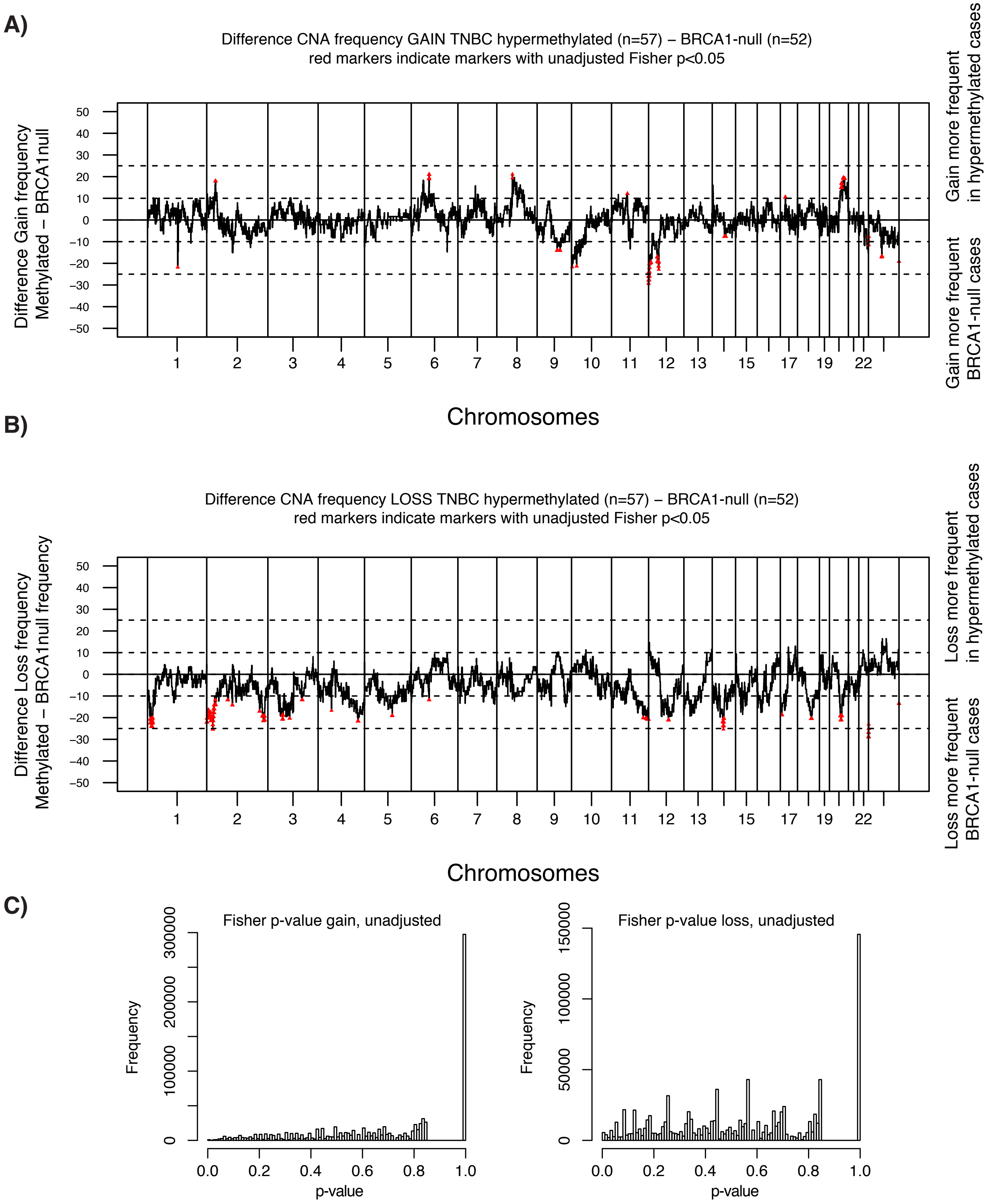

Supplementary Figure 4. Copy number differences between hypermethylated and BRCA1-null cases based on the combined 109-sample WGS cohort. (**A**) Difference in copy number gain frequency between hypermethylated minus BRCA1-null, i.e. a positive value above 0 means a higher frequency of gain in hypermethylated cases compared to BRCA1-null. (**B**) Difference in copy number loss between hypermethylated minus BRCA-null cases, i.e. a positive value above 0 means a higher frequency of loss in hypermethylated cases compared to BRCA1-null, whereas a negative value means more loss in BRCA1-null cases. (**C**) Histogram of uncorrected Fisher's exact p-values for each marker for copy number gain and loss respectively. P-values reported are two-sided.

In A and B, red triangles indicate which of the 931851 tested markers that have an uncorrected Fisher's exact p-value <0.05. No marker had a fdr adjusted p-value < 0.05. Fisher's tests were performe for gain and loss separately in 2x2 table format.

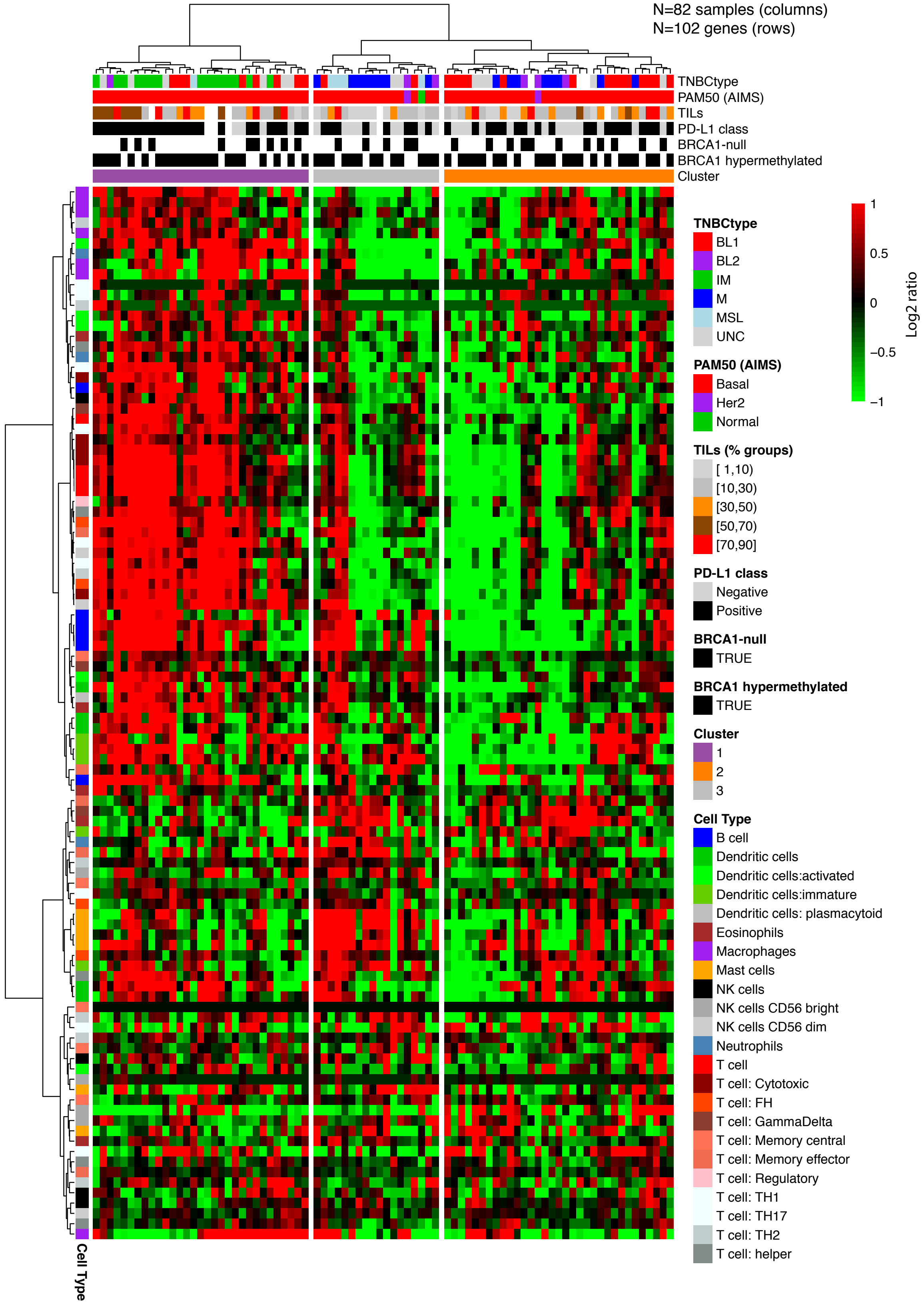

Supplementary Figure 5. Gene expression patterns of immune associated genes in *BRCA1*-null and hypermethylated SCAN-B cases. 102 genes associated with different immune cell types (see legend) were clustered using Pearson correlation and ward.D linkage in the 82 SCAN-B cases (25 *BRCA1*-null, 57 *BRCA1* hypermethylated) using mean-centered gene expression after offset of +0.1 followed by log2 transformation. Gene symbols are available in the Source Data file.

Supplementary Methods

Patient cohort and selection

The patient cohort in this study has previously been described [1]. Briefly, during September 1 2010 to March 31 2015, 408 patients were diagnosed with TNBC in the Region Skåne healthcare area based on data from the Swedish national breast cancer quality registry (NKBC). To derive this patient set the following exclusion criteria were used in a two-step fashion:

1: Removing non-TNBC cases

- Cases that were not ER-negative
- Cases that were not PR-negative
- Cases that were not HER2-negative

2: Removing TNBC cases with unclear treatment history

- Cases with no planned surgery were removed.
- Cases that did not have indication of planned pre- or postoperative treatment were removed.

Criteria 2 above excluded TNBC patients with an unclear/unknown treatment status based on registry data, irrespective of the type of treatment given. This meant that the identified and retained patients could have had neoadjuvant treatment, adjuvant systemic treatment, no treatment, or even palliative treatment due to metastatic disease already at time of diagnosis (thus including these patient categories in subsequent cohorts). Of the 408 patients, 340 provided informed written consent and were enrolled in the Sweden Cancerome Analysis – Breast (SCAN-B) study[2, 3] (ClinicalTrials.gov ID NCT02306096). The final tally of 254 samples were selected into this study based on also having available quality-controlled RNA sequencing (RNAseq) data from SCAN-B, sufficient DNA, and passing extensive review of available clinical data from individual patient´s files by a senior oncologist. RNAseq data for primary cases has been deposited in GEO series GSE96058 based on a previous study (outlining quality control filters and details of the RNAseq analysis). The 254 patients were diagnosed at any of the four main hospitals in the Region Skåne healthcare region, with a catchment area of approximately 1.3 million inhabitants (year 2017).

Of the 254 cases, 237 had successful whole genome sequencing performed as outlined in Staaf et al. [1], representing the final patient cohort from which *BRCA1* hypermethylated and *BRCA1* biallelic / germline inactivated cases (*BRCA1*-null) were selected. For the latter, the outlined scheme in Staaf et al. was used for defining a *BRCA1*-null phenotype.

Tissue sampling, DNA and RNA extraction

Fresh tumor tissue samples preserved in RNAlater (Qiagen, Hilden, Germany) were obtained in conjunction with routine clinical sampling by a diagnostic pathologist in regional pathology departments (see [3] for outline). RNA and DNA were extracted using the Qiagen Allprep extraction kit (Qiagen) as described[2]. DNA from whole blood was extracted by the Labmedicin Skåne Biobank, Lund, Sweden.

Prior germline testing and classification of *BRCA1* and *BRCA2* germline variants

46 patients had prior clinical genetic counseling involving NGS-based screening of *BRCA1* and *BRCA2*, or were enrolled in the SWEA research study (The Swedish BRCA1 and BRCA2

study collaborators (SWE-BRCA) Extended Analysis) for high-risk patients and screened for an extended panel of susceptibility genes. The inclusion criteria for the SWEA study were in line with the Swedish national clinical practice guidelines for breast cancer. Briefly, genetic testing was offered when there was at least a 10 % probability to detect a pathogenic germline variant in *BRCA1* or *BRCA2*, based on the patient's age at diagnosis, histology, and family history. Detected germline variants were classified according to the ENIGMA BRCA1/2 Gene variant Classification Criteria (2017-06-29) https://enigmaconsortium.org/library/general-documents/. Only class 5 variants were considered as pathogenic, corresponding to nine *BRCA1* and three *BRCA2* variants.

DNA promoter methylation analysis by pyrosequencing - Tumor DNA

Bisulfite conversion of genomic tumor DNA was performed with the column based EpiTecht Fast DNA Bisulfite kit (Qiagen GmBH, Hilden, Germany. 500 ng commercially available unmethylated and methylated DNA controls (Human Methylated & Non-methylated DNA Set, Zymo Research) were included in each bisulfite conversion run. After conversion, a methylation specific PCR was performed on control samples. PCR products were next analyzed on agarose gels to verify that bands were observed for the positive (methylated) control but not for the negative (unmethylated) control. The converted control samples were then included in the corresponding pyrosequencing run as controls. Promoter methylation analysis was performed using a PSQ MD 96 pyrosequencing instrument (Qiagen). The PyroMark analysis program was used for data analysis and all electropherograms were manually checked. For *BRCA1*, analysis was performed as originally described by [4], and included analysis of two CpG island regions. A 7% cut-off was used as reported [1]. CpG allele methylation percentage was averaged across each primer set and next merged to the mean of the two sets. Cut-offs were applied for making a call on methylation or no methylation.

DNA promoter methylation analysis by pyrosequencing - Blood DNA

Pyrosequencing analysis of BRCA1 promoter methylation levels was also performed in peripheral blood DNA from in total 104 of the 237 cases. The same primers as for tumor analyses were used. Bisulfite conversion was performed using the EZ-96 DNA Methylation Kit (Zymo Research, Irvine, CA, USA). 500 ng commercially available unmethylated and methylated DNA controls (Human Methylated & Non-methylated DNA Set, Zymo Research) were included in each bisulfite conversion run. After conversion, a methylation specific PCR was performed on control samples, with primers versus DPAK1 (included in the kits). PCR products were next analyzed on agarose gels to verify that bands were observed for the positive (methylated) control but not for the negative (unmethylated) control. The converted control samples were then included in the corresponding pyrosequencing run as controls. Promoter methylation analysis was performed using the same PSQ MD 96 pyrosequencing instrument as for tumors. Commercially available controls included in the bisulfite conversion were included in each pyrosequencing run as quality controls. The PyroMark analysis program was used for data analysis and all electropherograms were manually checked.

Global DNA methylation analysis

Global DNA methylation profiles of 235 SCAN-B TNBCs were successfully completed using the Illumina MethylationEPIC beadchips according to manufacturer's instructions, performed

at the Center for Translational Genomics, Lund University, Medicon Village, Lund. Among the analyzed cases were 57 *BRCA1* hypermethylated and 25 *BRCA1*-null cases. Preprocessing was performed according to the following steps:

1. IDAT files were loaded into the minfi R Bioconductor package ver 1.32.0 [5]. Functional normalization was performed using the "preprocessFunnorm" function, Beta values were derived using the "funnorm" function.
2. Probes flagged as poor-performing by Zhou et al. [6] were removed.
3. Correction for Infinium I/II probe bias as outlined [7, 8].

Preprocessing left 760405 probes for further analysis. Filtering for CpGs reduced the final dataset to 614977 probes. Briefly, the filtering was set so that, per CpG, there had to be an absolute difference in beta-value of at least 0.1 between the sample with the 5th lowest beta and the sample with the 5th highest beta in the 235 cases with DNA methylation data. In practice, this filter removes CpGs with a close to zero standard deviation in beta-value, that are uninformative in downstream supervised/unsupervised analyses.

Gene expression analyses

Gene expression data was available from Gene Expression Omnibus [9], series GSE96058, reported elsewhere [10] along with quality control and preprocessing information. FPKM data for specific genes were extracted and log2 transformed. For TNBCtype, IC10, CIT classification, 228 cases were available for analysis based on GSE96058 [10] (primary tumors only). For remaining cases, these were included separately and subtyped only using AIMS [11] (as this is a single sample predictor of molecular subtype) and analyzed for individual FPKM gene expression.

Classification according to different molecular subgroups in breast cancer was performed as follows, after *i*) an offset of +1 was added to all FPKM values, *ii*) log2 transformation:

- *PAM50*. PAM50 subtypes were obtained using the AIMS single sample classifier[11], based on the aims R package ver 1.18.0. All samples were classified.
- *TNBCtype* [12, 13]. For TNBCtype classification the entire GSE96058 data set was used. Data was mean-centered across all samples for each gene, TNBC cases were extracted and uploaded as a separate data set into the web-based classifier [13]. For a few cases the web-based application called the samples as not being ER-negative. These samples were removed from the TNBC data set (inferring missing values) and remaining samples were again uploaded to the web-based application for subtyping.
- *IC10*[14]. For IC10 classification the entire GSE96058 data set was used. Data was mean-centered across all samples for each gene. IC10 subgroups were obtained through the ic10 R package ver. 1.5 using default processing.
- *CIT*[15]. CIT subtypes were obtained through the citbcmst R package, using pearson correlation as distance method and gene symbol as matching entity.

Consensus clustering

Consensus clustering was performed in R [16] using the ConsensusClusterPlus R-package [17] ver. 1.50.0. For FPKM data as input this was first offset by +0.1, log2 transformed, and mean-centered across samples for each RefSeq associated gene. A filter step based on standard deviation of expression was used as defined in result presentations. In the clustering, we used Pearson correlation as distance metric and ward.D2 linkage. Additional parameters were pItem=0.8, pFeature=0.8, number of iterations = 2000.

Supervised Significance of Microarray analysis (SAM)
SAM analysis was performed using the functions in the siggenes R package ver 1.58.0. Analysis was performed using the sam() function with settings: rand = 123, control=samControl(n.delta=10000,q.version = 1,lambda = seq(0, 0.95, 0.01))). Analysis was performed on the same gene set as the unsupervised clustering.

Immune cell type deconvolution

To understand the immune cell composition of the TNBC samples (n=235 of the 237 samples used), we used two different approaches to explore the underlying distribution of the immune cells in tumors.

At first, we used Illumina EPIC array-based DNA methylation data for immune cell type deconvolution using EpiDISH with Robust Partial Correlations (RPC) as the method [18]. Here, we used two different references for deconvolution process, first one being DNase Hypersensitive Site (DHS) based curated CpGs for seven immune cell types (Monocytes, Neutrophils, Eosinophils, $CD4^+$ T-cells, $CD8^+$ T-cells, $CD56^+$ Natural Killer (NK) cells and B-cells) ("centDHSbloodDMC.m") and the other being similarly curated non-DHS CpGs based ("centBloodSub.m"). For further details regarding these references please refer to the original paper [18]. Estimates from both references were very similar for almost all cell types and in the final analysis the centBloodSub.m was used.

Next, we used mRNA expression from the matching tumor samples (n=235) to deconvolute the tumor microenvironment first using xCell [19] which digitally deconvolves the tumor samples into a wide range of possible cell types. Here, the number of reference cell types is quite high, so there is a small possibility that some unrelated cell types might show positive estimate by chance. Hence, in order to get closest possible to the ground truth, we downloaded single cell RNA Sequencing (scRNA-Seq) based mRNA expression dataset from an earlier study on primary TNBC [20] from GEO (GSE75688) and used the identified immune and non-immune cell types to do the deconvolution of the bulk tumor RNA-Seq data using CIBERSORTx [21]. CIBERSORTx is a modified version of earlier cell type deconvolution method CIBERSORT [22] with the option of constructing custom reference signature matrix using scRNA-Seq based tumor mRNA expression. We used the aforementioned scRNA-Seq based TNBC dataset for building the signature matrix and using that deconvolved the Lund TNBC tumors into 5 different cell types (Epithelial, stroma, macrophage, B and T cells) using default settings.

Survival analyses

Definition of clinical endpoints:

- Overall survival was obtained from national registries, calculated as the time from diagnosis to death of any cause.
- Invasive disease-free survival (IDFS) was defined according to STEEP guidelines[23], as the time from diagnosis to either death of any cause or invasive breast-cancer related events (loco-regional and distant recurrence).
- Distant relapse-free interval (DRFI) was defined according to STEEP guidelines as the time from surgery to diagnosis a distant relapse (event) or to last day of follow-up (censoring). Events include patients that first developed a loco-regional relapse, and then a distant relapse. For these patients the day of the distant relapse was used.

Exclusion criteria for outcome analyses:

- Neoadjuvant treatment

- Metastatic disease at time of diagnosis (including microinvasive disease).
- Metastatic disease identified immediately prior to, or during adjuvant chemotherapy.
- Patients not managed in an adjuvant setting (irrespective if adjuvant treatment or not provided later).
- Bilateral breast cancer.
- Lost to follow-up before start of systemic treatment.
- Unclear histological type (one case).
- For DRFI, patients with a relapse or death from a malignancy of uncertain origin were excluded. These patients were however included in OS and IDFS analyses.

Multivariable analyses

Analysis was performed using the coxph R function from the survival R package ver 3.1-12. Covariates in multivariable Cox regression were patient age (<40, 40-60, ≥60 years), lymph node status (N0/N+), tumor size (≤20, >20mm), and tumor grade (1,2,3). Data for lymph node status, tumor size and tumor grade were obtained from [1].

Statistical analyses

All p-values reported from statistical tests are two-sided if not otherwise specified. Box-plot elements corresponds to: i) center line = median, ii) box limits = upper and lower quartiles, iii) whiskers = 1.5x interquartile range.

Whole Genome Sequencing Analysis

Whole genome sequencing analysis is extensively described in Staaf et al. [1]. Processed data for the 237 final cases from that study was used. Corresponding meta WGS data was obtained from the study reported by Nik-Zainal et al. [24] for a set of *BRCA1*-null cases. For analyses of drivers (mutations and copy number) we restricted the analysis to the drivers defined in Nik-Zainal et al. using the supplementary information provided in that study as starting point (Supplementary Table 14 in Nik-Zainal et al.) combined with driver data from Staaf et al. [1]. For mutational and rearrangement signatures supplemental data from Nik-Zainal was used and combined with SCAN-B data

For general comparisons of tumor cell content in SCAN-B samples we used WGS estimates based on the ASCAT algorithm. For the specific comparison of pyrosequencing methylation % versus tumor cell content for SCAN-B samples we used estimates from the Battenberg algorithm (https://github.com/cancerit/cgpBattenberg) which can account for subclones.

HRD classification

HRD classification was obtained from [1], based on two different classifiers; HRDetect [25] and genomic scars (copy number based) [26] computed from WGS data. To note, for each classification one sample was classified as HRD-low, however not the same sample. For HRDetect, the HRD-low hypermethylated case showed concurrent MMRd which caused the algorithm to inaccurately classify the sample as HRD-low.

Neoantigen prediction from substitutions

The NeoPredPipe software (https://github.com/MathOnco/NeoPredPipe) was used to predict putative neoantigens with substitution mutation calls provided by CaVEMan (https://cancerit.github.io/CaVEMan/) and HLA typing provided by the Polysolver software. As input, hg19 was used as the human reference genome throughout all analysis for the neoantigen predictions. NeoPredPipe was run with default parameters except that options "-c 1 2 -m" where set. Polysolver was run on WGS data from blood DNA with options "unknown ethnicity", "use population-level allele frequencies as priors", and "do not use empirical insert size distribution". Only variants with a PASS flag in the variant call file from CaVEMan was used as input to NeoPredPipe. Integration with RNAseq expression was done as outlined for NeoPredPipe, and only neoantigens with an expression >0.1 was kept. The NeoPredPipe version available in the GitTrunk Feb 7 2020 was used, referenced as https://github.com/MathOnco/NeoPredPipe/tree/3384e75634c564b961ba2a65ac66905d9117d3b9, which is an improved version of the tagged 1.1 version on GitHub.

Supplementary References

1. Staaf J*, et al.* Whole-genome sequencing of triple-negative breast cancers in a population-based clinical study. *Nature medicine* **25**, 1526-1533 (2019).

2. Saal LH*, et al.* The Sweden Cancerome Analysis Network - Breast (SCAN-B) Initiative: a large-scale multicenter infrastructure towards implementation of breast cancer genomic analyses in the clinical routine. *Genome Med* **7**, 20 (2015).

3. Ryden L*, et al.* Minimizing inequality in access to precision medicine in breast cancer by real-time population-based molecular analysis in the SCAN-B initiative. *Br J Surg* **105**, e158-e168 (2018).

4. Saal LH*, et al.* Recurrent gross mutations of the PTEN tumor suppressor gene in breast cancers with deficient DSB repair. *Nature genetics* **40**, 102-107 (2008).

5. Aryee MJ*, et al.* Minfi: a flexible and comprehensive Bioconductor package for the analysis of Infinium DNA methylation microarrays. *Bioinformatics*, (2014).

6. Zhou W, Laird PW, Shen H. Comprehensive characterization, annotation and innovative use of Infinium DNA methylation BeadChip probes. *Nucleic acids research* **45**, e22 (2017).

7. Holm K*, et al.* An integrated genomics analysis of epigenetic subtypes in human breast tumors links DNA methylation patterns to chromatin states in normal mammary cells. *Breast Cancer Res* **18**, 27 (2016).

8. Karlsson A*, et al.* Genome-wide DNA methylation analysis of lung carcinoma reveals one neuroendocrine and four adenocarcinoma epitypes associated with patient outcome. *Clin Cancer Res* **20**, 6127-6140 (2014).

9. Gene Expression Omnibus. [cited 2019; Available from: http://www.ncbi.nlm.nih.gov/geo/

10. Brueffer C*, et al.* Clinical Value of RNA Sequencing–Based Classifiers for Prediction of the Five Conventional Breast Cancer Biomarkers: A Report From the Population-Based Multicenter Sweden Cancerome Analysis Network—Breast Initiative. *JCO Precision Oncology*, 1-18 (2018).

11. Paquet ER, Hallett MT. Absolute assignment of breast cancer intrinsic molecular subtype. *Journal of the National Cancer Institute* **107**, 357 (2015).

12. Lehmann BD*, et al.* Identification of human triple-negative breast cancer subtypes and preclinical models for selection of targeted therapies. *J Clin Invest* **121**, 2750-2767 (2011).

13. Chen X*, et al.* TNBCtype: A Subtyping Tool for Triple-Negative Breast Cancer. *Cancer Inform* **11**, 147-156 (2012).

14. Ali HR*, et al.* Genome-driven integrated classification of breast cancer validated in over 7,500 samples. *Genome biology* **15**, 431 (2014).

15. Guedj M*, et al.* A refined molecular taxonomy of breast cancer. *Oncogene* **31**, 1196-1206 (2012).

16. The R Project for Statistical Computing. [cited 2016; Available from: www.r-project.org

17. Wilkerson MD, Hayes DN. ConsensusClusterPlus: a class discovery tool with confidence assessments and item tracking. *Bioinformatics* **26**, 1572-1573 (2010).

18. Teschendorff AE, Breeze CE, Zheng SC, Beck S. A comparison of reference-based algorithms for correcting cell-type heterogeneity in Epigenome-Wide Association Studies. *BMC Bioinformatics* **18**, 105 (2017).

19. Aran D, Hu Z, Butte AJ. xCell: digitally portraying the tissue cellular heterogeneity landscape. *Genome biology* **18**, 220 (2017).

20. Chung W*, et al.* Single-cell RNA-seq enables comprehensive tumour and immune cell profiling in primary breast cancer. *Nat Commun* **8**, 15081 (2017).

21. Newman AM*, et al.* Determining cell type abundance and expression from bulk tissues with digital cytometry. *Nature biotechnology* **37**, 773-782 (2019).

22. Newman AM*, et al.* Robust enumeration of cell subsets from tissue expression profiles. *Nature methods* **12**, 453-457 (2015).

23. Hudis CA*, et al.* Proposal for standardized definitions for efficacy end points in adjuvant breast cancer trials: the STEEP system. *J Clin Oncol* **25**, 2127-2132 (2007).

24. Nik-Zainal S*, et al.* Landscape of somatic mutations in 560 breast cancer whole-genome sequences. *Nature* **534**, 47-54 (2016).

25. Davies H*, et al.* HRDetect is a predictor of BRCA1 and BRCA2 deficiency based on mutational signatures. *Nature medicine* **23**, 517-525 (2017).

26. Telli ML*, et al.* Homologous Recombination Deficiency (HRD) Score Predicts Response to Platinum-Containing Neoadjuvant Chemotherapy in Patients with Triple-Negative Breast Cancer. *Clin Cancer Res* **22**, 3764-3773 (2016).
