# Supplementary material for: Comprehensive molecular comparison of BRCA1 hypermethylated and BRCA1 mutated triple negative breast cancers
Source: Nat Commun. 2020 Jul 27;11:3747. doi: 10.1038/s41467-020-17537-2 (PMC7385112; doi:10.1038/s41467-020-17537-2)
Supplement: Supplementary file 3 — Description of Additional Supplementary Files [file 41467_2020_17537_MOESM3_ESM.pdf]

Description of Additional Supplementary Files

File Name: Supplementary Data 1
Description: Table providing detailed clinicopathological and molecular information and results for hypermethylated and *BRCA1*-null SCAN-B cases.

File Name: Supplementary Data 2
Description: Table providing gene expression, copy number and DNA methylation data on eight differentially expressed genes.
