# Supplementary material for: Comprehensive molecular comparison of BRCA1 hypermethylated and BRCA1 mutated triple negative breast cancers
Source: Nat Commun. 2020 Jul 27;11:3747. doi: 10.1038/s41467-020-17537-2 (PMC7385112; doi:10.1038/s41467-020-17537-2)
Supplement: Supplementary file 6 — Reporting Summary [file 41467_2020_17537_MOESM6_ESM.pdf]

nature research

Corresponding author(s): Johan Staaf

Last updated by author(s): Jun 12, 2020

Reporting Summary

Nature Research wishes to improve the reproducibility of the work that we publish. This form provides structure for consistency and transparency in reporting. For further information on Nature Research policies, see Authors & Referees and the Editorial Policy Checklist.

Statistics

For all statistical analyses, confirm that the following items are present in the figure legend, table legend, main text, or Methods section.

n/a	Confirmed	
☐	☒	The exact sample size (n) for each experimental group/condition, given as a discrete number and unit of measurement
☐	☒	A statement on whether measurements were taken from distinct samples or whether the same sample was measured repeatedly
☐	☒	The statistical test(s) used AND whether they are one- or two-sided<br>*Only common tests should be described solely by name; describe more complex techniques in the Methods section.*
☐	☒	A description of all covariates tested
☐	☒	A description of any assumptions or corrections, such as tests of normality and adjustment for multiple comparisons
☐	☒	A full description of the statistical parameters including central tendency (e.g. means) or other basic estimates (e.g. regression coefficient) AND variation (e.g. standard deviation) or associated estimates of uncertainty (e.g. confidence intervals)
☐	☒	For null hypothesis testing, the test statistic (e.g. F, t, r) with confidence intervals, effect sizes, degrees of freedom and P value noted<br>*Give P values as exact values whenever suitable.*
☒	☐	For Bayesian analysis, information on the choice of priors and Markov chain Monte Carlo settings
☒	☐	For hierarchical and complex designs, identification of the appropriate level for tests and full reporting of outcomes
☒	☐	Estimates of effect sizes (e.g. Cohen's d, Pearson's r), indicating how they were calculated

Our web collection on statistics for biologists contains articles on many of the points above.

Software and code

Policy information about availability of computer code

Data collection

Global DNA methylation profiles of 235 SCAN-B TNBCs were successfully completed using the Illumina MethylationEPIC beadchips according to manufacturer's instructions, performed at the Center for Translational Genomics, Lund University, Medicon Village, Lund. Among the analyzed cases were 57 BRCA1 hypermethylated and 25 BRCA1-null cases.

BRCA1 blood DNA hypermethylation status was analyzed using pyrosequencing, similar to tumor cases previously described (Staaf et al. Nature Medicine 2019).

WGS and RNAseq was performed as previously outlined (Staaf et al. Nature Medicine 2019).

Tumor infiltrating lymphocytes were analyzed on whole slide FFPE sections. PD-L1 IHC was performed on tissue microarrays using the Roche SP-142 antibody according to Swedish pathology guidelines and evaluated by an experienced breast cancer pathologist.

Data analysis

Reference annotations relates to references in the Supplementary Methods document attached with the submission.

1. Pyrosequencing: The PyroMark analysis program associated with the Qiagen instrument was used for data analysis and all electropherograms were manually checked.

2. Global DNA methylation analysis: Preprocessing was performed according to the following steps:
- IDAT files were loaded into the minfi R Bioconductor package ver 1.32.0 (ref 8). Functional normalization was performed using the "preprocessFunnorm" function, Beta values were derived using the "funnorm" function.
- Probes flagged as poor-performing by Zhou et al. (ref 9) were removed.
- Correction for Infinium I/II probe bias as outlined (ref 10, 11).

3. Gene expression: Preprocessing is described in Staaf et al. Nature Medicine 2019. From this study we extracted subtype classifications. All unsupervised analysis was performed in R ver 3.6.1 (ref 17) using the ConsensusClusterPlus R-package ver 1.50.0 (ref 18). For FPKM

data as input this was first offset by +0.1, log2 transformed, and mean-centered across samples for each RefSeq associated gene. A filter step based on standard deviation of expression was used as defined in result presentations. In the clustering we used Pearson correlation as distance metric and ward.D2 linkage. Additional parameters were pItem=0.8, pFeature=0.8, number of iterations = 2000.

4. Immune cell deconvolution:
To understand the immune cell composition of the TNBC samples (n=235 of the 237 samples used), we used two different approaches to explore the underlying distribution of the immune cells in tumors. At first, we used Illumina EPIC array-based DNA methylation data for immune cell type deconvolution using EpiDISH with Robust Partial Correlations (RPC) as the method (ref 19). Here, we used two different references for deconvolution process, first one being DNase Hypersensitive Site (DHS) based curated CpGs for seven immune cell types (Monocytes, Neutrophils, Eosinophils, CD4+ T-cells, CD8+ T-cells, CD56+ Natural Killer (NK) cells and B-cells) ("centDHSbloodDMC.m") and the other being similarly curated non-DHS CpGs based ("centBloodSub.m"). For further details regarding these references please refer to the original paper (ref19). Estimates from both references were very similar for almost all cell types and in the final analysis the centBloodSub.m was used. Next, we used mRNA expression from the matching tumor samples (n=235) to deconvolute the tumor microenvironment first using xCell (ref 20) which digitally deconvolves the tumor samples into a wide range of possible cell types. Here, the number of reference cell types is quite high, so there is a small possibility that some unrelated cell types might show positive estimate by chance. Hence, in order to get closest possible to the ground truth, we downloaded single cell RNA Sequencing (scRNA-Seq) based mRNA expression dataset from an earlier study on primary TNBC (ref 21) from GEO (GSE75688) and used the identified immune and non-immune cell types to do the deconvolution of the bulk tumor RNA-Seq data using CIBERSORTx (ref 22). CIBERSORTx is a modified version of earlier cell type deconvolution method CIBERSORT (ref 23) with the option of constructing custom reference signature matrix using scRNA-Seq based tumor mRNA expression. We used the aforementioned scRNA-Seq based TNBC dataset for building the signature matrix and using that deconvolved the Lund TNBC tumors into 5 different cell types (Epithelial, stroma, macrophage, B and T cells) using default settings. The following software versions were used for the analyses:
R-packages:
* EpiDISH_2.0.2 (For DNA methylation based immune cell-type deconvolution)
* scales_1.0.0 (Used to rescale output values from xCell to a scale of 0-100)

Web cell-type deconvolution tools used:
xCell : https://xcell.ucsf.edu/
CIBERSORTx: https://cibersortx.stanford.edu/

5. Survival analyses:
Definition of clinical endpoints:
- Overall survival was obtained from national registries, calculated as the time from diagnosis to death of any cause.
- Invasive disease-free survival (IDFS) was defined according to STEEP guidelines (ref 24), as the time from diagnosis to either death of any cause or invasive breast-cancer related events (loco-regional and distant recurrence).
- Distant relapse-free interval (DRFI) was defined according to STEEP guidelines as the time from surgery to diagnosis a distant relapse (event) or to last day of follow-up (censoring). Events include patients that first developed a loco-regional relapse, and then a distant relapse. For these patients the day of the distant relapse was used.

Exclusion criteria for outcome analyses:
- Neoadjuvant treatment
- Metastatic disease at time of diagnosis (including microinvasive disease).
- Metastatic disease identified immediately prior to, or during adjuvant chemotherapy.
- Patients not managed in an adjuvant setting (irrespective if adjuvant treatment or not provided later).
- Bilateral breast cancer.
- Lost to follow-up before start of systemic treatment.
- Unclear histological type (one case).
- For DRFI, patients with a relapse or death from a malignancy of uncertain origin were excluded. These patients were however included in OS and IDFS analyses.

Multivariable analyses
Analysis was performed using the coxph R function from the survival R package ver 3.1-12. Covariates in multivariable Cox regression were patient age (<50, ≥50 years), lymph node status (N0/N+), tumor size (≤20, >20mm), and tumor grade (1,2,3). Data for lymph node status, and tumor size were obtained from NKBC data. Tumor grade was obtained from clinical review of individual patient's files.

6. Statistical analyses
All p-values reported from statistical tests are two-sided if not otherwise specified. Box-plot elements corresponds to: i) center line = median, ii) box limits = upper and lower quartiles, iii) whiskers = 1.5x interquartile range.

7. Whole Genome Sequencing Analysis
Whole genome sequencing analysis is extensively described in Staaf et al. (ref 6). Processed data for the 237 final cases from that study was used. Corresponding meta WGS data was obtained from the study reported by Nik-Zainal et al. (ref 25) for a set of BRCA1-null cases. For analyses of drivers (mutations and copy number) we restricted the analysis to the drivers defined in Nik-Zainal et al. using the supplementary information provided in that study as starting point (Supplementary Table 14 in Nik-Zainal et al.) combined with driver data from Staaf et al. 1. For mutational and rearrangement signatures supplemental data from Nik-Zainal was used and combined with SCAN-B data. For general comparisons of tumor cell content in SCAN-B samples we used WGS estimates based on the ASCAT algorithm. For the specific comparison of pyrosequencing methylation % versus tumor cell content for SCAN-B samples we used estimates from the Battenberg algorithm (https://github.com/cancerit/cgpBattenberg) which can account for subclones obtained from ref 6. Detailed settings for the Battenberg algorithm is available from ref 25.

8. HRD classification

HRD classification was obtained from 6, based on two different classifiers; HRDetect (ref 26) and genomic scars (copy number based) (ref 27) computed from WGS data.

9. Neoantigen prediction from substitutions
The NeoPredPipe software (https://github.com/MathOnco/NeoPredPipe) was used to predict putative neoantigens with substitution mutation calls provided by CaVEMan (https://cancerit.github.io/CaVEMan/) and HLA typing provided by the Polysolver software. As input, hg19 was used as the human reference genome throughout all analysis for the neoantigen predictions. NeoPredPipe was run with default parameters except that options "-c 1 2 -m" where set. Polysolver was run on WGS data from blood DNA with options "unknown ethnicity", "use population-level allele frequencies as priors", and "do not use empirical insert size distribution". Only variants with a PASS flag in the variant call file from CaVEMan was used as input to NeoPredPipe. Integration with RNAseq expression was done as outlined for NeoPredPipe, and only neoantigens with an expression >0.1 was kept. The NeoPredPipe version available in the GitTrunk Feb 7 2020 was used, referenced as https://github.com/MathOnco/NeoPredPipe/tree/3384e75634c564b961ba2a65ac66905d9117d3b9, which is an improved version of the tagged 1.1 version on GitHub.

For manuscripts utilizing custom algorithms or software that are central to the research but not yet described in published literature, software must be made available to editors/reviewers. We strongly encourage code deposition in a community repository (e.g. GitHub). See the Nature Research guidelines for submitting code & software for further information.

Data

Policy information about availability of data

All manuscripts must include a data availability statement. This statement should provide the following information, where applicable:

- Accession codes, unique identifiers, or web links for publicly available datasets
- A list of figures that have associated raw data
- A description of any restrictions on data availability

Data access to the original data taken from our previous study is outlined in detail in Staaf et al. Nat Med 2019. This include detailed complete somatic DNA mutation data. Illumina DNA methylation data for BRCA1-deficient cases (hypermethylated and BRCA1-null) and for the additional BRCA1 gene expression profiled samples (expression counts, not sequence due to patient consent) are available from GSE148748 at Gene Expression Omnibus (Illumina DNA methylation) and DOI: 10.17632/2dbh285999.1 (FPKM gene expression additional BRCA1 cases).

Data availability statement
For BRCA1 hypermethylated and BRCA1-null SCAN-B cases mapping of clinical and molecular classifications to gene expression data GSE96058 [https://www.ncbi.nlm.nih.gov/geo/query/acc.cgi?acc=GSE96058] is available in Supplementary Data 1. Illumina DNA methylation data for BRCA1 hypermethylated and BRCA1-null SCAN-B cases is available as GSE148748 [https://www.ncbi.nlm.nih.gov/geo/query/acc.cgi?acc=GSE148748] at Gene Expression Omnibus. FPKM RNAseq data for the additional 27 BRCA1-mutated cases from Jönsson et al. 23 is available in an online repository associated with this study [http://dx.doi.org/10.17632/2dbh285999.1]. Source data are provided with this paper. The source data underlying figures 2A-D, 3A-D, 4A, 4D-G, 5A-D, and 6A-B are provided as a Source Data file.

Field-specific reporting

Please select the one below that is the best fit for your research. If you are not sure, read the appropriate sections before making your selection.

☒ Life sciences ☐ Behavioural & social sciences ☐ Ecological, evolutionary & environmental sciences

For a reference copy of the document with all sections, see nature.com/documents/nr-reporting-summary-flat.pdf

Life sciences study design

All studies must disclose on these points even when the disclosure is negative.

Sample size

Sample size was determined based on availability of samples in our previous WGS analyzed cohort (Staaf et al. Nature Medicine 2019). Thus, no sample-size collection analysis was performed, as we included all samples with multi-layer omics data.

Data exclusions

Data was collected from our previous study in Nature Medicine (Staaf et al. 2019) (n=237). For two samples we could not perform Illumina EPIC DNA methylation analyses due to lack of DNA. These were however not BRCA1-deficient (the focus of the study). Moreover, for a number of BRCA1-deficient cases we were not able to collect their corresponding clinical FFPE block for detailed in situ analyses (PD-L1 TMA staining and whole-slide TILs analysis). In all instances, we report the exact number of cases analyzed by respective method.

Replication

Replication was not performed for genomic analyses (WGS, RNAseq, DNA methylation), i.e. WGS was performed once for a sample. The reason for this was due to cost and availability of starting material (DNA and RNA).

Data for tumor BRCA1 promoter hypermethylation was obtained from our previous study (Staaf et al. Nat Med 2019). Thus these experiments were not re-performed (i.e. no replication).

Replication was performed for the first set of 36 cases in the BRCA1 blood DNA promoter hypermethylation analyses. For these cases, experiments were later replicated together with an additional second validation series to ensure complete agreement in kit reagent lot numbers, PCR settings, and instrument settings. The reason for replication of these samples was to ensure a uniform experimental setting, as hypermethylation values were at the limit of detection of the pyrosequencing instrument, and experimental conditions (PCR formats, bisulfite conversion kits etc) did not allow all samples to be processed in one run.

Randomization	Randomization was not applicable to this study. The study population used in the current study originates from a population-based observational cohort study in south Sweden (SCAN-B, see Ryden et al. Br J Surg 105, e158-e168 (2018)).
Blinding	Blinding was not applicable to this study. The patient cohort was defined in a previous study (Staaf et al. Nature Medicine, 2019).

Reporting for specific materials, systems and methods

We require information from authors about some types of materials, experimental systems and methods used in many studies. Here, indicate whether each material, system or method listed is relevant to your study. If you are not sure if a list item applies to your research, read the appropriate section before selecting a response.

Materials & experimental systems

n/a	Involved in the study	
☐	☒	Antibodies
☒	☐	Eukaryotic cell lines
☒	☐	Palaeontology
☒	☐	Animals and other organisms
☐	☒	Human research participants
☐	☒	Clinical data

Methods

n/a	Involved in the study	
☒	☐	ChIP-seq
☒	☐	Flow cytometry
☒	☐	MRI-based neuroimaging

Antibodies

Antibodies used	The Roche SP142 PD-L1 antibody was used according to manufacturer's recommendations on a Ventana instrument (Roche) .
Validation	The SP142 is a commercially available antibody that has been extensively validated by Roche.

Human research participants

Policy information about studies involving human research participants

Population characteristics	Patients recruited to the SCAN-B observational cohort, on which study is based, is recruited in a population-based setting. Approximately 85-90% of ALL breast cancer patients with primary disease is enrolled in SCAN-B at the active sites (see Ryden et al. Br J Surg 105, e158-e168 (2018)). This means that there is no real cohort bias, and that research cohorts can be shown to mimic actual population-based cohorts. For this study we selected patients were analyzed by RNAseq and WGS from our previous study (Staaf et al. Nature Medicine 2019). In that study we demonstrate that the actual study population is representative of the background population in the healthcare region the patients were recruited from.
Recruitment	Patients were recruited to the SCAN-B observational cohort as described in previous studies (Ryden et al. Br J Surg 105, e158-e168 (2018). Selection of patients for this study was based on available multi-layer omics data from our previous study on the cohort (Staaf et al. Nature Medicine 2019).
Ethics oversight	The Regional Ethical Review Board in Lund, Sweden approved the SCAN-B study. Relevant ethical approval numbers related to this specific study is 2009/658, 2010/383, 2012/58, 2016/742, 2018/267, and 2019/01252.

Note that full information on the approval of the study protocol must also be provided in the manuscript.

Clinical data

Policy information about clinical studies

All manuscripts should comply with the ICMJE guidelines for publication of clinical research and a completed CONSORT checklist must be included with all submissions.

Clinical trial registration	ClinicalTrials.gov ID NCT02306096
Study protocol	SCAN-B is an observational cohort study. Thus there is no specific study protocol related to patient therapy as study inclusion does not affect patient therapy in any way.
Data collection	Patients were recruited between September 1 2010 to March 31 2015 in the Skåne Healthcare region, Sweden. In this healthcare region four hospitals were active sites for SCAN-B recruitment during the time period: Lund, Malmö, Helsingborg, Kristianstad. For included patients, we performed a detailed review of patient charts.
Outcomes	Survival outcomes were assessed based on data in patient charts. Exclusion criteria for survival analysis is detailed above and in our previous study (Staaf et al. Nature Medicine 2019).
